# Adherence to the low-fat diet pattern reduces the risk of lung cancer in American adults aged 55 years and above: a prospective cohort study

**DOI:** 10.1016/j.jnha.2024.100240

**Published:** 2024-04-24

**Authors:** Linglong Peng, Qingqing Du, Ling Xiang, Haitao Gu, Haoyun Luo, Zhiquan Xu, Hongmei He, Boning Xia, Zhihang Zhou, Yaxu Wang, Ying Chen

**Affiliations:** a Department of Gastrointestinal Surgery, The Second Affiliated Hospital of Chongqing Medical University, Chongqing, China; b Department of Clinical Nutrition, The Second Affiliated Hospital of Chongqing Medical University, Chongqing, China; c Department of Gastroenterology, The Second Affiliated Hospital of Chongqing Medical University, Chongqing, China; d Health Medicine Center, The Second Affiliated Hospital of Chongqing Medical University, Chongqing, China

**Keywords:** Low-fat diet, Lung cancer, Epidemiology, Cohort study, Cancer prevention

## Abstract

**Objectives:**

There is little evidence on the association between low-fat dietary patterns and lung cancer risk among middle-aged and older adults. To fill this gap, we comprehensively investigated the association of adherence to a low-fat diet (LFD) and intake of different fat components including saturated, monounsaturated, and polyunsaturated fatty acids with incidence of lung cancer and its subtypes [non-small cell lung cancer (NSCLC) and small cell lung cancer (SCLC)] among adults aged 55 years and older.

**Design:**

A prospective cohort study with a mean follow-up time of 8.8 years.

**Setting and participants:**

This study used data from the Prostate, Lung, Colorectal, and Ovarian (PLCO) Cancer Screening Trial. The study population included 98,459 PLCO participants age 55 and over at baseline who completed food frequency questionnaires providing detailed dietary information and had no history of cancer.

**Methods:**

Dietary intake was assessed using a validated food frequency questionnaire at baseline. A LFD score was calculated based on fat, protein, and carbohydrate intake as a percentage of total calories. Cox proportional hazards regression models were used to estimate hazard ratios (HRs) and 95% confidence intervals (CIs) for the association between LFD score and intake of fat components (in quartiles) and incident lung cancer and its subtypes over follow-up. Restricted cubic spline analyses were conducted to examine possible nonlinear relationships. Subgroup analyses were performed to evaluate potential effect modifiers, and several sensitivity analyses were conducted to assess the stability of the findings.

**Results:**

During a follow-up of 869,807.9 person-years, 1,642 cases of lung cancer were observed, consisting of 1,408 (85.75%) cases of NSCLC and 234 (14.25%) cases of SCLC. The highest versus the lowest quartiles of the LFD score were found to be associated with a reduced risk of lung cancer (HR, 0.76; 95% CI, 0.66−0.89), NSCLC (HR, 0.79; 95% CI, 0.67−0.93), and SCLC (HR, 0.59; 95% CI, 0.38−0.92). The restricted cubic spline plots demonstrated a linear dose-response relationship between the LFD score and the risk of lung cancer as well as its subtypes. This risk reduction association for overall lung cancer was more pronounced in smokers (HR, 0.71; 95% CI, 0.60−0.84; P for interaction = 0.003). For fat components, high consumption of saturated fatty acids was associated with an increased lung cancer risk (HR, 1.35; 95% CI, 1.10–1.66), especially for SCLC (HR, 2.05; 95% CI, 1.20–3.53). No significant association was found between consumption of monounsaturated or polyunsaturated fatty acids and incident lung cancer and its subtypes.

**Conclusions:**

Our findings suggest that adherence to LFD may reduce the lung cancer risk, particularly in smokers; while high saturated fatty acids consumption may increase lung cancer risk, especially for SCLC, among middle-aged and older adults in the US population.

## 1 Introduction

Lung cancer remains the leading cause of cancer mortality worldwide, with over 2 million new cases and 1.8 million deaths reported in 2020 [1]. Cigarette smoking is the primary risk factor for lung cancer, but lung cancer development and progression also involve multiple other factors including genetics, environmental exposures, and diet [[2], [3], [4]]. Given the high disease burden of lung cancer, identifying potentially modifiable risk factors such as dietary factors could inform prevention efforts and provide new avenues for risk reduction.

Recent studies have examined associations between specific dietary components and lung cancer risk. An analysis of the UK Biobank showed higher lung cancer risk with consumption of red and processed meats and a Western diet pattern, while fruits, vegetables, and fiber were associated with lower risk [5]. A large European cohort study identified correlations between intake of 92 dietary factors and lung cancer risk. Higher consumption of fiber, vitamin C, and fruits was linked to lower risk, while higher intake of retinol, offal, and beer/cider was associated with higher risk [6]. In summary, current evidence indicates low-fat foods high in fruits and fibers may be protective against lung cancer, while high-fat foods like red and processed meats appear to increase lung cancer risk.

A low-fat diet (LFD) limits total fat intake to reduce calories and prevent chronic diseases [7]. Inverse associations have been found between total fat intake and risk of breast [8], pancreatic [9], and skin [10] cancers in studies defining LFD as less than 30% of calories from fat pancreatic, and skin cancer risk. However, defining LFD solely by total fat may not reflect real-world eating habits. Thus, a novel LFD score was developed based on percentage of calories from fat, protein, and carbohydrates, with higher scores indicating greater LFD adherence [11]. Our previous study using this LFD score found a 55% lower liver cancer risk in the highest versus lowest LFD score quartile [12]. However, the direct association between a low-fat dietary pattern as a whole and lung cancer risk has not yet been explored. To bridge this knowledge gap, we conducted this prospective study using data from the Prostate, Lung, Colorectal, and Ovarian (PLCO) Cancer Screening Trial.

## 2 Methods

### 2.1 Study population

To determine if screening tests could reduce colorectal, lung, prostate, and ovarian cancer-related mortalities, the PLCO Cancer Screening Trial, a randomized, controlled trial was performed by National Cancer Institute (NCI), USA. The PLCO trial design and methodology have been reported previously [13,14]. Nearly 155,000 participants between 55–74 years were recruited between November 1993–July 2001 by ten screening centers across the USA. The data on cancer diagnoses until 2009 and the patient’s mortality until 2018 were collected, with a median follow-up duration of 11.3 and 19.2 years, respectively. The data on all participant’s demographics and medical history were collected by distributing a baseline questionnaire (BQ). Additionally, a dietary questionnaire like the Dietary History Questionnaire (DHQ) and a Supplemental Questionnaire (SQX) were also used to collect participant diet-related data. The PLCO study protocol was approved by the Institutional Review Boards of all study centers and NCI. All participants provided written informed consent. For this analysis, we used publicly available data sets approved by the NCI (Project ID: PLCO-1399). Hence, no ethical approval was required regarding the inclusion of human participants.

We aimed to analyze the association between LFD and the risk of lung cancer and its subtypes. Therefore, the participants were excluded following the following criteria: (i) Failure to return BQ (n = 4918); (ii) participants with invalid DHQ [including lack of eight + frequency of responses on DHQ, excessive intake of calories (the first and last percentile) for both genders assessed using DHQ, and availability of DHQ completion date prior to death] (n = 38462); (iii) participants diagnosed with any cancer prior to DHQ completion (n = 9684); (iv) participants with outcome events including lung cancer diagnosis, deceased, or lost during follow-up between randomization and completion of DHQ (n = 68); (v) participants with inconsistent daily calorie intake like women with a calorie intake “<600 or >3500 kcal/day,” and men with “<800 or >4200 kcal/day” (n = 3296) [11]. Finally, we included 98,459 participants in this study (Supplementary Fig. S1).

### 2.2 Assessments of LFD scores

We calculated the LFD scores following previously reported criteria [11]. Briefly, the participants were divided into 11 strata based on the percent of the energy gained from macronutrients like fat, carbohydrates, and protein consumption (Supplementary Table S1). For fat, 10 points were assigned to the participants in the lowest stratum and 0 points to participants in the highest stratum. The order of the strata was reversed for macronutrients like protein and carbohydrates. Subsequently, we summarized the points for the three macronutrients for calculating LFD scores for all participants, ranging from 0–30. Thus, a higher LFD score indicated better adherence by the participants to the LFD pattern. Specifically, we extracted the percent of energy gained from three macronutrients from the DHQ. The raw responses in DHQ have been processed into analysis-ready variables like as daily food frequency, pyramid service, and gram intake with the aid of the “DietCalc “software, the “US National Dietary data survey nutrient” and the “Nutrition Data Systems for Research” databases. The reliability of evaluating participants’ diet and nutrition data by DHQ was reported elsewhere [15,16].

### 2.3 Confirmation of lung cancer diagnosis

In the PLCO cancer screening trial, the cancer diagnosis was confirmed using the annual research update mailed to the living participants. The relevant medical record abstract form was to record data on cancer diagnosis collected from participants. For patients with confirmed primary lung cancer diagnosis, additional information on the procedures used for diagnosis, the stage, grade, histopathology type of cancer, and initial cancer treatment was recorded by the participant’s physicians. We extracted the information on the diagnosis date, type like SCLC and NSCLC, as well as ICD-O-2 code from the PLCO data. The endpoint was lung cancer and its subtypes incidence.

### 2.4 Arranging covariates

Baseline covariates like the participant’s sex (male, female), level of education (college below, college graduate, postgraduate), height, race (white, non-white), weight, smoking (never, current or former), use of aspirin (no, yes), history of diabetes, emphysema, and family history of lung cancer (no, yes) were obtained from BQ. Body mass index (BMI) was calculated as weight (kg)/height (m^2^). Additionally, DHQ was used to collect data on the participant’s age, alcohol consumption (no, yes), the consumption of dietary constituents such as cholesterol, sodium, potassium, magnesium, calcium, and phosphorus, the pyramid food consumption (total fruits, vegetables, lean meat, dairy, and added sugars), energy intake from diet, and the consumption of total carbohydrates, fats, and protein. The summarized minutes of moderate to strenuous activity/week were referred to as physical activity obtained from SQX.

### 2.5 Statistical analysis

During data processing, we identified a few varying degrees of missing variables, which could affect the stability and accuracy of subsequent analysis. Therefore, the missing components of variables were handled based on absent variables. For less than 5% missing values, the missing values of the continuous variables, like BMI, were supplemented by its median value; and categorical variables such as the level of education, familial history of cancer, the use of aspirin use, and history of emphysema and diabetes were complemented by the modal value. Additionally, for the variable of physical activity level with more than 25% missing values, the multiple imputation method was applied. Supplementary Table S2 shows detailed information on the imputation data.

To investigate the potential correlation of the LFD score with lung cancer and its subtypes risk, the Cox proportional hazards regression model was used for calculating the hazard ratios (HRs) and 95% confidence intervals (CIs). The model was adjusted for potential confounders, and the follow-up period was utilized as the time metric. In this study, we defined the follow-up time as the DHQ completion date to lung cancer occurrence, death, or loss during or the end of follow-up (i.e., December 31, 2009), whichever occurred first (Supplementary Fig. S2). All participants were split into quartiles based on the LFD score, and the first quarter (lowest LFD score) was the reference group. Based on the follow-up time, the person-years of each quartile were quantified. Schoenfeld residuals approach was used to judge whether the LFD score is a time-varying variable. We conducted a trend analysis of lung cancer and its subtypes risk using the Cox regression model by assigning the median value of each quartile of DRRD scores to all individuals within that quartile and treating it as a continuous variable. To control for potential confounding factors, we employed two models with multiple variables in the Cox regression analyses. Model 1 was adjusted for participant age, race, and sex, while model 2 additionally adjusted for education level, family history of lung cancer, alcohol consumption, BMI, aspirin use, physical activity, history of emphysema and diabetes, and energy intake from diet. Additionally, we utilized the restricted cubic spline model to assess the potential nonlinear association of LFD score with lung cancer and its subtypes risk. The association of dietary fatty acids with the risk of lung cancer and its subtypes was also assessed via the above-mentioned methods including covariates selection and establishment of the Cox regression model. Specially, dietary fatty acids were categorized into quartiles, with the lowest quartile as the reference group.

We conducted a subgroup analysis to investigate the relationship between LFD score and overall lung cancer risk, taking into account potential confounders such as participant age, sex, BMI, drinking or smoking status, and history of emphysema or diabetes. The P-values for interaction were calculated to determine the significance of these potential confounders. Additionally, we performed sensitivity analyses to assess the robustness of our primary analyses. These included (1) repeated analysis on participants without any missing data; (2) exclusion of participants with a history of emphysema or diabetes; (3) exclusion of participants from the initial two and four years of follow-up; and 4) further adjustment for pyramid food consumption and dietary constituent intake variables.

The descriptive statistics are expressed as mean ± standard deviation and number (percentage) for continuous and categorical variables. All statistical analyses were performed using the R 4.1.1 software and a two-tailed P < 0.05 indicated the significance level.

## 3 Results

### 3.1 Characteristics of the study population

98459 participants with mean [standard deviation (SD)] age of 65.5 (5.73) were included in the study, of which 47,218 (47.96%) were males. The characteristics of participants based on the quartiles of LFD score at baseline are shown in Table 1. The mean (SD) LFD score of all participants was 15.00 (6.27) and a higher quartile of the LFD scores indicates higher adherence to LFD. Among the LFD scores, the participants in the highest quartile were older, female, and non-white, and had a higher level of educational attainment compared to those in the lowest quartile. Regarding lifestyle and medical history, participants in the highest versus the lowest quartiles had a higher physical activity level; but had low BMI, family history of lung cancer, and history of emphysema, consumed alcohol, and were smokers. Regarding food consumption, the intake of fruits, vegetables, and dairy was higher, and the intake of lean meat, added sugars, and energy consumption was lower among participants in the highest quartile. With regards to the intake of nutritional constituents, the intake of potassium, magnesium, calcium, phosphorus, carbohydrate, and protein was higher, and the intake of cholesterol, sodium, and fat was lower among participants in the highest compared to the lowest quartile.

**Table 1:** Baseline characteristics of study population according to quartiles of low-fat diet scores.

		Quartiles of low-fat diet scores			
Characteristics	Overall	Quartile 1 (≤10)	Quartile 2 (11−15)	Quartile 3 (16−20)	Quartile 4 (≥21)
Number of participants	98459	26715	26151	24764	20829
Low-fat diet score	15.00 ± 6.27	7.33 ± 2.40	12.98 ± 1.42	18.03 ± 1.43	23.75 ± 2.36
Age	65.52 ± 5.73	65.08 ± 5.63	65.24 ± 5.67	65.73 ± 5.77	66.19 ± 5.79
Sex					
Male	47218 (47.96%)	14771 (55.29%)	13807 (52.80%)	11267 (45.50%)	7373 (35.40%)
Female	51241 (52.04%)	11944 (44.71%)	12344 (47.20%)	13497 (54.50%)	13456 (64.60%)
Race					
White	91221 (92.65%)	25092 (93.92%)	24419 (93.38%)	22404 (90.47%)	19306 (92.69%)
Non-white	7238 (7.35%)	1623 (6.08%)	1732 (6.62%)	2360 (9.53%)	1523 (7.31%)
Education level					
College below	62599 (63.58%)	17938 (67.15%)	16900 (64.62%)	15571 (62.88%)	12190 (58.52%)
College graduate	17353 (17.62%)	4486 (16.79%)	4585 (17.53%)	4334 (17.50%)	3948 (18.95%)
Postgraduate	18507 (18.80%)	4291 (16.06%)	4666 (17.84%)	4859 (19.62%)	4691 (22.52%)
Body mass index (kg/m^2^)	27.20 ± 4.79	27.58 ± 4.86	27.47 ± 4.75	27.04 ± 4.72	26.57 ± 4.74
Family history of lung cancer					
No	85845 (87.19%)	23116 (86.53%)	22731 (86.92%)	21643 (87.40%)	18355 (88.12%)
Yes	10266 (10.43%)	2887 (10.81%)	2778 (10.62%)	2535 (10.24%)	2066 (9.92%)
Possibly	2348 (2.38%)	712 (2.67%)	642 (2.45%)	586 (2.37%)	408 (1.96%)
Smoker					
Never	47233 (47.97%)	10720 (40.13%)	11892 (45.47%)	12775 (51.59%)	11846 (56.87%)
Current or former	51226 (52.03%)	15995 (59.87%)	14259 (54.53%)	11989 (48.41%)	8983 (43.13%)
Drinker					
No	26681 (27.10%)	6023 (22.55%)	6388 (24.43%)	7259 (29.31%)	7011 (33.66%)
Yes	71778 (72.90%)	20692 (77.45%)	19763 (75.57%)	17505 (70.69%)	13818 (66.34%)
Aspirin use					
No	52242 (53.06%)	14308 (53.56%)	13781 (52.70%)	13220 (53.38%)	10933 (52.49%)
Yes	46217 (46.94%)	12407 (46.44%)	12370 (47.30%)	11544 (46.62%)	9896 (47.51%)
History of emphysema					
No	96410 (97.92%)	25959 (97.17%)	25584 (97.83%)	24299 (98.12%)	20568 (98.75%)
Yes	2049 (2.08%)	756 (2.83%)	567 (2.17%)	465 (1.88%)	261 (1.25%)
History of diabetes					
No	91990 (93.43%)	25028 (93.69%)	24365 (93.17%)	23139 (93.44%)	19458 (93.42%)
Yes	6469 (6.57%)	1687 (6.31%)	1786 (6.83%)	1625 (6.56%)	1371 (6.58%)
Physical activity (min/week)	123.28 ± 108.79	109.11 ± 103.00	119.60 ± 106.95	128.15 ± 109.94	140.29 ± 114.11
Energy intake from diet (kcal/day)	1728.71 ± 658.04	1936.85 ± 722.85	1785.82 ± 657.33	1634.18 ± 603.05	1502.45 ± 529.56
Pyramid food consumption					
Total fruits (servings/day)	2.74 ± 1.99	1.89 ± 1.35	2.47 ± 1.63	3.22 ± 2.29	3.62 ± 2.19
Total vegetables (servings/day)	3.90 ± 2.24	3.72 ± 2.01	3.84 ± 2.11	3.87 ± 2.30	4.24 ± 2.58
Total lean meat (oz./day)	7.20 ± 4.30	8.17 ± 4.60	7.94 ± 4.62	6.48 ± 3.90	5.90 ± 3.35
Total dairy (servings/day)	1.37 ± 1.11	1.21 ± 0.97	1.34 ± 1.07	1.33 ± 1.09	1.69 ± 1.25
Added Sugars (tsp/day)	12.43 ± 8.77	12.98 ± 8.59	12.77 ± 8.67	13.08 ± 10.40	10.52 ± 6.41
Nutrition constituent intake					
Cholesterol(mg/day)	207.53 ± 127.19	269.60 ± 152.36	228.66 ± 122.19	175.48 ± 97.62	139.49 ± 72.63
Sodium (mg/day)	2728.47 ± 1126.48	2954.08 ± 1182.28	2834.60 ± 1150.77	2571.90 ± 1082.10	2491.99 ± 993.21
Potassium (mg/day)	3250.60 ± 1185.26	3108.40 ± 1126.18	3223.43 ± 1163.69	3244.48 ± 1204.41	3474.38 ± 1229.79
Magnesium (mg/day)	322.09 ± 119.36	317.56 ± 122.28	320.95 ± 118.09	317.60 ± 117.30	334.66 ± 118.71
Calcium (mg/day)	749.65 ± 387.80	695.28 ± 354.04	736.13 ± 378.47	733.38 ± 383.09	855.72 ± 424.55
Phosphorus (mg/day)	1148.23 ± 470.02	1147.55 ± 474.05	1162.15 ± 475.69	1103.76 ± 461.66	1184.48 ± 463.36
Total Carbohydrate (% energy)	51.99 ± 9.36	43.36 ± 6.86	49.37 ± 6.04	56.42 ± 7.29	61.10 ± 5.89
Total fat (% energy)	31.78 ± 7.52	39.44 ± 6.15	33.61 ± 4.13	28.47 ± 4.27	23.59 ± 3.97
Total protein (% energy)	15.44 ± 2.93	14.34 ± 2.72	15.48 ± 2.89	15.28 ± 3.02	16.98 ± 2.39

Values are means (standard deviation) for continuous variables and percentages for categorical variables.

### 3.2 Low-fat diet, dietary fatty acids and the risk of lung cancer and its subtypes

**Fig. 1:**
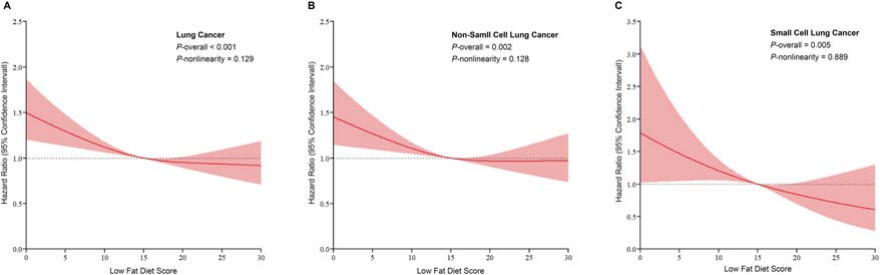
Dose–response analyses on the association of low-fat diet scores with the risk of lung cancer (A), non-small cell lung cancer (B) and small cell lung cancer (C). Hazard ratios were adjusted for age (years), sex (male, female), race (white, non-white), educational level (college below, college graduate, postgraduate), body mass index (kg/m^2^), family history of lung cancer (no, yes, possibly), smoker (never, current or former), drinker (no, yes), aspirin use (no, yes), history of emphysema (no, yes), history of diabetes (no, yes), physical activity (min/week), and energy intake from diet (kcal/day).

During the mean (SD) follow-up duration of 8.83 (1.93) years, corresponding to 869,807.9 person-years, we observed 1642 patients with lung cancer, including 1408 (85.75%) patients with NSCLC and 234 (14.25%) patients with SCLC. In the fully multivariable-adjusted model, the participants in the highest quartile had a reduced risk of lung cancer compared to the lowest quartile (HR _Q4 vs. Q1_ = 0.76, 95% CI: 0.66−0.89, *P* < 0.001 for trend) (Table 2). Additionally, an inverse association was observed between the LFD scores and the risk of NSCLC (HR _Q4 vs. Q1_ = 0.79, 95% CI: 0.67−0.93, *P* = 0.001 for trend) as well as SCLC (HR _Q4 vs. Q1_ = 0.59, 95% CI: 0.38−0.92, *P* = 0.013 for trend) (Table 2). The restricted cubic spline analysis confirmed the linearity assumptions between the LFD score and the risk of lung cancer, NSCLC, and SCLC. Fig. 1 depicts an inverse dose-response association between the LFD score and lower risks of lung cancer (*P* = 0.129 for nonlinearity), NSCLC (*P* = 0.128 for nonlinearity), and SCLC (*P* = 0.889 for nonlinearity) in a linear pattern.

**Table 2:** Association of low-fat diet scores with the risk of lung cancer and its subtypes.

	No. of	No. of		Hazard ratio (95% confidence interval)		
Quartiles of low-fat diet score	Participants	Cases	Person-years	Unadjusted	Model 1^a^	Model 2^b^
Lung cancer						
Quartile 1 (≤10)	26715	576	231834.4	1.00 (reference)	1.00 (reference)	1.000 (reference)
Quartile 2 (11–15)	26151	456	229364.0	0.80 (0.71, 0.90)	0.80 (0.70, 0.90)	0.89 (0.79, 1.01)
Quartile 3 (16–20)	24764	351	220352.9	0.64 (0.56, 0.73)	0.63 (0.55, 0.72)	0.78 (0.68, 0.89)
Quartile 4 (≥21)	20829	259	188256.6	0.55 (0.48, 0.64)	0.55 (0.47, 0.64)	0.76 (0.66, 0.89)
*P* for trend				<0.001	<0.001	<0.001
Non-small cell lung cancer						
Quartile 1 (≤10)	26667	486	231734.1	1.00 (reference)	1.000 (reference)	1.000 (reference)
Quartile 2 (11–15)	26063	387	228870.8	0.81 (0.70, 0.92)	0.80 (0.70, 0.92)	0.89 (0.78, 1.02)
Quartile 3 (16–20)	24712	303	220118.0	0.65 (0.57, 0.75)	0.65 (0.60, 0.75)	0.78 (0.68, 0.91)
Quartile 4 (≥21)	20783	232	187939.0	0.58 (0.50, 0.68)	0.58 (0.50, 0.68)	0.79 (0.67, 0.93)
*P* for trend				<0.001	<0.001	0.001
Small cell lung cancer						
Quartile 1 (≤10)	26370	91	230614.5	1.00 (reference)	1.00 (reference)	1.00 (reference)
Quartile 2 (11–15)	25720	68	226979.9	0.76 (0.55, 1.04)	0.76 (0.55, 1.04)	0.89 (0.65, 1.22)
Quartile 3 (16–20)	24438	48	218657.0	0.55 (0.39, 0.79)	0.56 (0.39, 0.79)	0.76 (0.53, 1.09)
Quartile 4 (≥21)	20523	27	186414.7	0.36 (0.24, 0.56)	0.36 (0.24, 0.56)	0.59 (0.38, 0.92)
*P* for trend				<0.001	<0.001	0.013

a Adjusted for age (years), sex (male, female) and race (white, non-white).

b Adjusted for model 1 plus educational level (college below, college graduate, postgraduate), body mass index (kg/m^2^), family history of lung cancer (no, yes, possibly), smoker (never, current or former), drinker (no, yes), aspirin use (no, yes), history of emphysema (no, yes), history of diabetes (no, yes), physical activity (min/week), and energy intake from diet (kcal/day).

Regards to dietary fatty acids, we observed a significant positive association between saturated fatty acids (SFA) intake and the risks of lung cancer (HR _Q4 vs. Q1_ = 1.35, 95% CI: 1.10–1.66, *P* = 0.007 for trend) and SCLC (HR _Q4 vs. Q1_ = 2.05, 95% CI: 1.20–3.53, *P* = 0.005 for trend) in the fully adjusted model (Table 3). However, we did not observe any significant association between the risks of lung cancer, NSCLC, and SCLC and intake of polyunsaturated fatty acids (PUFA) (Supplementary Table S3) or monounsaturated fatty acids (MUFA) (Supplementary Table S4) in all crude and adjusted models.

**Table 3:** Association of saturated fatty acids with the risk of lung cancer and its subtypes.

	No. of	No. of		Hazard ratio (95% confidence interval)		
Quartiles of SFA (g/day)	Participants	Cases	Person-years	Unadjusted	Model 1^a^	Model 2^b^
Lung Cancer						
Quartile 1 (≤11.99)	24655	338	220101.1	1.00 (reference)	1.00 (reference)	1.00 (reference)
Quartile 2 (12.00−17.34)	24594	395	218060.5	1.18 (1.02, 1.37)	1.17 (1.01, 1.35)	1.14 (0.98, 1.33)
Quartile 3 (17.35−25.04)	24624	420	217150.7	1.26 (1.09, 1.46)	1.23 (1.06, 1.42)	1.20 (1.02, 1.42)
Quartile 4 (≥25.05)	24586	489	214495.5	1.49 (1.30, 1.71)	1.42 (1.23, 1.64)	1.35 (1.10, 1.66)
*P* for trend				<0.001	<0.001	0.007
Non-Small Cell Lung Cancer						
Quartile 1 (≤11.98)	24570	299	219512.1	1.00 (reference)	1.00 (reference)	1.00 (reference)
Quartile 2 (11.99−17.33)	24543	348	217804.9	1.17 (1.01, 1.37)	1.16 (0.99, 1.36)	1.15 (0.98, 1.35)
Quartile 3 (17.34−25.02)	24571	366	216894.5	1.24 (1.07, 1.45)	1.20 (1.03, 1.41)	1.20 (1.01, 1.43)
Quartile 4 (≥25.03)	24541	395	214450.4	1.36 (1.17, 1.58)	1.29 (1.10, 1.51)	1.27 (1.01, 1.59)
*P* for trend				<0.001	0.003	0.066
Small Cell Lung Cancer						
Quartile 1 (≤11.98)	24309	38	218273.1	1.00 (reference)	1.00 (reference)	1.00 (reference)
Quartile 2 (11.99−17.33)	24242	47	216187.6	1.25 (0.82, 1.92)	1.24 (0.81, 1.90)	1.16 (0.75, 1.80)
Quartile 3 (17.34−25.02)	24259	54	215275.7	1.45 (0.95, 2.19)	1.42 (0.93, 2.17)	1.29 (0.81, 2.03)
Quartile 4 (≥25.03)	24241	95	212929.6	2.57 (1.77, 3.75)	2.54 (1.71, 3.76)	2.05 (1.20, 3.53)
*P* for trend				<0.001	<0.001	0.005

a Adjusted for age (years), sex (male, female) and race (white, non-white).

b Adjusted for model 1 plus educational level (college below, college graduate, postgraduate), body mass index (kg/m^2^), family history of lung cancer (no, yes, possibly), smoker (never, current or former), drinker (no, yes), aspirin use (no, yes), history of emphysema (no, yes), history of diabetes (no, yes), physical activity (min/week), and energy intake from diet (kcal/day).

### 3.3 Subgroup and sensitivity analyses

**Fig. 2:**
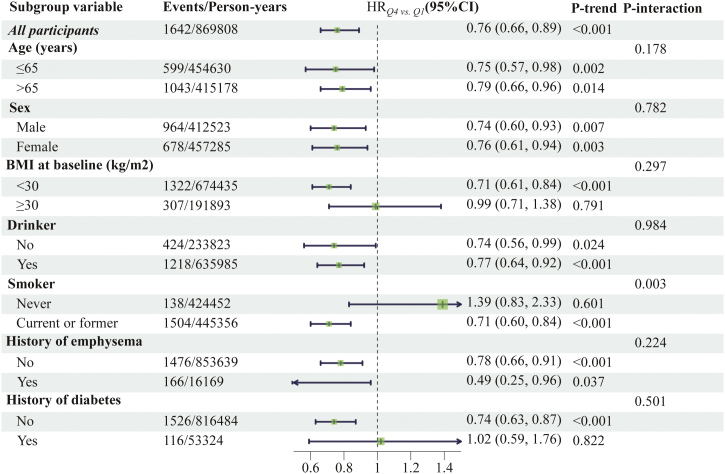
Subgroup analyses on the association of low-fat diet scores with the risk of overall lung cancer. Hazard ratios were adjusted for age (years), sex (male, female), race (white, non-white), educational level (college below, college graduate, postgraduate), body mass index (kg/m^2^), family history of lung cancer (no, yes, possibly), smoker (never, current or former), drinker (no, yes), aspirin use (no, yes), history of emphysema (no, yes), history of diabetes (no, yes), physical activity (min/week), and energy intake from diet (kcal/day). Hazard ratios were not adjusted for the stratification factor.

As per the results of subgroup analyses, the participant’s age, sex, BMI, drinking status, history of emphysema or diabetes, did not affect the association between the LFD score and lung cancer risk (all *P* > 0.05 for interaction) (Fig. 2). However, significant interactions between smoking status and LFD score were observed (*P* for interaction = 0.003). The inverse association between LFD score with the incidence of overall lung cancer was more pronounced in current or former smokers (HR _Q4vs. Q1_ = 0.71, 95% CI: 0.60−0.84, *P* < 0.001 for trend) than in never smokers (HR _Q4vs. Q1_ = 1.39, 95% CI: 0.83–2.33, *P* = 0.601 for trend) (Fig. 2). In the sensitivity analyses, the primary association was similar on repeated analysis of participants without missing data, and excluding participants with a history of emphysema or diabetes, and excluding lung cancer cases during 2-year or 4-year follow-up at baseline, and further adjustment for pyramid food consumption and intake of nutrition constituents at baseline, which highlights that the robustness of the association between the LFD score and lung cancer risk (Supplementary Table S5).

## 4 Discussion

In our study, we examined the relationship between adherence to LFD pattern and lung cancer risk in middle-aged and older adults in the US. We found a significant inverse association between LFD adherence and lung cancer incidence, including both NSCLC and SCLC. This association remained robust after adjusting for confounders and was confirmed through sensitivity analyses. Notably, a linear dose-dependent relationship was observed, indicating that individuals following LFD pattern had a lower risk of lung cancer. Moreover, the protective effect of LFD adherence was particularly pronounced among smokers. Conversely, high consumption of SFA was associated with an increased risk of lung cancer, especially SCLC. Overall, our study underscores the potential role of dietary patterns, specifically LFD, in reducing the risk of lung cancer, while emphasizing the need for further research to validate these findings and inform public health interventions.

Generally, the LFD is defined as a dietary pattern that restricts dietary fat intake to less than 30% of total daily caloric intake [17]. In this context, LFD is often recommended as a way to reduce the risk of certain chronic diseases [18,19]. Research on the epidemiology of tumors and LFD has mainly focused on several important clinical trials related to breast cancer [[20], [21], [22]]. The WHI-DM trial showed a significant decrease in the incidence of ER-positive and PR-negative breast cancer in the low-fat group during the intervention period, compared to the normal-diet group [20]. The WHEL trial analyzed the effects of LFD on previously treated patients with early breast cancer, and the results showed no significant benefits on patients with breast cancer [21]; but a significant improvement in the survival of breast cancer patients without hot flashes on LFD was observed [22]. The LFD typically emphasizes the consumption of low-fat foods, such as fruits, vegetables, and whole grains; while limiting or avoiding high-fat foods, such as fatty meats and full-fat dairy products [17]. However, human body obtains energy from the three main macronutrients present in food: fats, proteins, and carbohydrates. Therefore, relying solely on the proportion of energy derived from fats to evaluate the LFD presents some challenges and difficulties because this definition pattern for LFD may ignore the contributions of other macronutrients and there is virtually no food in reality that is solely composed of pure fat as an energy source. In our present study, we addressed a gap in the literature by investigating the association between the LFD pattern and lung cancer risk among middle-aged and older adults. Utilizing the LFD score method, our novel approach considered the energy proportions of the three macronutrients in food and the characteristics of low-fat energy supply [11], thereby overcoming the limitation of traditional method for evaluating LFD. Our results indicated an inverse association between the LFD score and the risk of lung cancer and its subtypes incidence in a non-linear dose-response manner after adjusting for potential confounders, contributing valuable evidence to the field.

Dietary fatty acids could be involved in the onset and progression of lung cancer [23]; however, the results of previous epidemiological studies are still inconsistent [24,25]. A recent meta-analysis performed on ten prospective cohort studies from Asia, the USA, and Europe determined the correlation between dietary fatty acids and the risk of lung cancer [26]. The results indicated a correlation between a high intake of total fat and SFA and an increased risk of lung cancer, specifically for SCLC. On the contrary, the results revealed an association between high PUFA intake and a reduced lung cancer risk. However, no significant association between MUFA and lung cancer risk was reported. In this study, while our primary focus was on examining the relationship between adherence to the LFD pattern and lung cancer risk, our investigation into specific types of dietary fatty acids further corroborates the conclusions drawn by Yang et al. The observed increase in lung cancer incidence among participants with high SFA intake, particularly for SCLC, underscores the importance of dietary fat composition in lung cancer etiology. Notably, our results align with the recent meta-analysis, providing additional evidence supporting the adverse effects of high SFA intake on lung cancer risk. Overall, our study adds valuable insights to the existing literature and reinforces the need for dietary interventions targeting fat intake to reduce the burden of lung cancer.

Smoking is the primary cause of lung cancer [27]. Smokers have a 4–10 times higher risk of developing lung cancer compared to non-smokers [28]. Therefore, we conducted a subgroup analysis of smokers and non-smokers and evaluated the interaction between smoking status and LFD on overall lung cancer risk. Our results suggest that adhering to LFD is more beneficial in preventing lung cancer in smokers than in non-smokers. A recent study analyzed the association between smoking status and dietary energy density (a marker for the quality of diet), and found that the diet quality of smokers was significantly poorer than that of non-smokers [29]. Specially, former smokers had better diet quality than current smokers, but still significantly lower than never smokers [29], indicating smoking significantly influences an individual's dietary habits, and individuals who quit smoking tend to adopt healthier diets, such as LFD. This may partly explain why LFD has a preventive effect against lung cancer in smokers. Additionally, the potential interaction between LFD adherence and smoking in the etiological pathway of lung cancer development should be considered. It is important to note that unexpected results in subgroup analyses cannot be ruled out. Therefore, large-sample, long-term follow-up studies are needed to verify our findings.

Numerous underlying mechanisms could be associated with the involvement of LFD score in lung cancer risk. First, high-fat diets directly alter the metabolism and the state cells from healthy tissues in animal models, thereby increasing their susceptibility to cancers [30]. High-fat diet activates lipid-ligand transcription factors like PPAR-δ, PPAR-α (primarily in the liver), and PPAR-γ (primarily in the adipose tissue) promote the oxidation of fatty acid, which regulates the storage of dietary fats and catabolism, thereby promoting the onset and progression of tumors [31]. Second, beyond their structural and metabolic roles, lipids also act as intra- and intercellular signaling molecules [32]. Cancer cells secrete these bioactive lipids to regulate various cellular processes and promote the occurrence and metastasis of cancer by altering the proliferation, invasion, and migration of cells and angiogenesis in an autocrine or paracrine manner [[33], [34], [35]]. Third, cell-cell interactions mediated by lipids in the tumor microenvironment could aid in creating conditions to promote the survival of cancer cells [36]. The adipocytes and stromal cells in tumors secrete fatty acids, which exert tumor-promoting effects by either damaging or enhancing immune cell function in the tumor microenvironment [37,38]. In the tumor microenvironment, lipids can damage natural killer cells and T-cell function and reduce the secretion of enzymes and cytokines to induce apoptosis [39,40]. Therefore, long-term consumption of a diet rich in fat increases the susceptibility to cancers.

Strengths of this study include its comprehensive methodology and robust findings. It is a prospective analysis conducted in a large population, providing substantial statistical power. The significant finding of a reduced risk of lung cancer associated with adherence to LFD pattern is noteworthy and contributes to the existing literature. Furthermore, the study demonstrates good robustness through multiple sensitivity analyses, affirming the reliability of the observed inverse association. The extensive adjustment for potential confounders, including demographic, lifestyle, and dietary factors, enhances the validity of the results and minimizes the influence of residual confounders. Overall, these strengths underscore the importance and credibility of the study's findings regarding the potential role of LFD in reducing the risk of lung cancer.

Our study has several limitations. Firstly, residual confusion cannot be fully ruled out, although we accounted for many potential confounding factors and a series of sensitivity analyses showed a high degree of stability of our results. Secondly, as with other nutritional epidemiologic studies, measurement errors in dietary assessment are a concern, although the questionnaires used in participating cohorts have been validated and shown good validity. Thirdly, we cannot assess the effects of possible dietary changes over time because only baseline dietary information in the PLCO trial is comprehensive and the subsequent supplementary dietary questionnaire contains missing dietary data. However, it should be noted that this single dietary assessment approach has been adopted by many cohort studies [12,41]. Additionally, the dietary habits of adults generally do not change easily during the follow-up period. Finally, all individuals included in our study were American adults aged 55–74 years; thus, our findings should be interpreted with caution for other populations.

## 5 Conclusions

In American middle-aged and older population, we have established an association of high LFD score with a decreased risk of developing lung cancer and its subtypes, particularly among individuals who are current and former smokers. Regards to dietary fatty acids, high consumption of SFA may contribute to an increased risk of lung cancer, with a higher risk observed for SCLC in particular. Therefore, our findings support the potential benefits of adhering to LFD and reducing SFA intake as a strategy for preventing lung cancer.

## Authors’ contributions

L.P. and Y.C. designed the research, applied for the original data, supervised the study, and revised the manuscript. L.P., Q.D., L.X., and Y.W. performed data collection, statistical analysis, and drafted the original manuscript. H.G., Z.X. and H.H. assisted with statistical methodology. H.L., B.X. and Z.Z. contributed to data visualization and manuscript revision. All authors reviewed and approved the final manuscript.

## Funding

This work was supported by the Natural Science Foundation Project of Chongqing, Chongqing Science and Technology Commission, China (grant numbers: cstc2021jcyj-msxmX0153 to Linglong Peng; cstc2021jcyj-msxmX0112 to Yaxu Wang; CSTB2022NSCQ-MSX1005 to Haitao Gu); the Kuanren Talents Project of the Second Affiliated Hospital of Chongqing Medical University, China (grant number: kryc-yq-2110 to Haitao Gu; 202417-48 to Linglong Peng); the Joint Medical Research Project of Science and Health Department, Nanan District, Chongqing, China (grant number: 2021-08 to Boning Xia); and the China Social Welfare Foundation Project (grant number: HLCXKT-20230137 to Ying Chen).

## Ethics approval and consent to participate

The PLCO trial was conducted in accordance with the Declaration of Helsinki principles. The study protocol received approval from the National Cancer Institute. All participants provided informed consent prior to enrollment in the trial.

## Availability of data and materials

The data supporting the findings of this study are available from the National Cancer Institute, but restrictions apply due to license limitations. The data were used under license approval for the current study and are not publicly available. However, the data can be obtained from the authors upon reasonable request and with permission from the National Cancer Institute.

## Competing interests

The authors declare that they have no competing interests.

## Appendix A Supplementary data

The flow chart of identifying eligible participants. PLCO, Prostate, Lung, Colorectal, and Ovarian; BQ, baseline questionnaire; DHQ, diet history questionnaire.

The timeline and follow-up scheme of our study.
