# Supplementary material for: Adherence to the low-fat diet pattern reduces the risk of lung cancer in American adults aged 55 years and above: a prospective cohort study
Source: J Nutr Health Aging. 2024 Apr 24;28(7):100240. doi: 10.1016/j.jnha.2024.100240 (PMC12433782; doi:10.1016/j.jnha.2024.100240)
Supplement: Supplementary file 1 [file mmc1.docx]

**Supplementary Table 1. Criteria for determining the low-fat diet scores**

**Supplementary Table 2. Distribution of variables with missing data before and after imputation**

|  | **Low-Fat Diet Scores** | | |
| --- | --- | --- | --- |
| Points | Total fat (% energy) | Total Carbohydrate (% energy) | Total protein (% energy) |
| 0 | ≥41.59 | ≤39.94 | ≤11.76 |
| 1 | 38.47–41.58 | 39.95–43.83 | 11.77–12.91 |
| 2 | 36.29–38.46 | 43.84–46.59 | 12.92–13.71 |
| 3 | 34.41–36.28 | 46.60–48.87 | 13.72–14.38 |
| 4 | 32.72–34.42 | 48.88–50.94 | 14.39–15.01 |
| 5 | 31.02–32.71 | 50.95–52.98 | 15.02–15.63 |
| 6 | 29.23–31.01 | 52.99–55.05 | 15.64–16.29 |
| 7 | 27.26–29.22 | 55.06–57.41 | 16.30–17.01 |
| 8 | 24.92–27.25 | 57.42–60.24 | 17.02–17.92 |
| 9 | 21.71–24.91 | 60.25–64.31 | 17.93–19.25 |
| 10 | ≤21.70 | ≥ 64.32 | ≥19.26 |

|  |  |  | No. (%) with |
| --- | --- | --- | --- |
| Variable | Before imputation | After imputation | missing data |
| Educational level |  |  | 196 (0.20%) |
| College below | 62403 (63.51%) | 62599 (63.58%) |  |
| College graduate | 17353 (17.66%) | 17353 (17.62%) |  |
| Postgraduate | 18507 (18.83%) | 18507 (18.80%) |  |
| Body mass index (kg/m^2^) | 27.21±4.82 | 27.20±4.79 | 1293 (1.31%) |
| Family history of lung cancer |  |  | 757 (0.77%) |
| No | 85088 (87.09%) | 85845 (87.19%) |  |
| Yes | 10266 (10.51%) | 10266 (10.43%) |  |
| Possibly | 2348 (2.40%) | 2348 (2.38%) |  |
| Aspirin use |  |  | 426 (0.43%) |
| No | 51816 (52.86%) | 52242 (53.06%) |  |
| Yes | 46217 (47.14%) | 46217 (46.94%) |  |
| History of emphysema |  |  | 515 (0.52%) |
| No | 95895 (97.91%) | 96410 (97.92%) |  |
| Yes | 2049 (2.09%) | 2049 (2.08%) |  |
| History of diabetes |  |  | 520 (0.53%) |
| No | 91470 (93.39%) | 91990 (93.43%) |  |
| Yes | 6469 (6.61%) | 6469 (6.57%) |  |
| Physical activity (min/week) | 125.15±123.32 | 123.28±108.79 | 24915 (25.30%) |

**Supplementary Table 3. Association of poly-unsaturated fatty acids with the risk of lung cancer and its subtypes**

|  | **No. of** | **No. of** |  | **Hazard ratio (95% confidence interval)** | | |
| --- | --- | --- | --- | --- | --- | --- |
| **Quartiles of PUFA (g/day)** | **Participants** | **Cases** | **Person-years** | **Unadjusted** | **Model 1^a^** | **Model 2^b^** |
| **Lung Cancer** |  |  |  |  |  |  |
| Quartile 1 (≤8.90) | 24630 | 419 | 218468.5 | 1.00 (reference) | 1.00 (reference) | 1.00 (reference) |
| Quartile 2 (8.91-12.58) | 24633 | 388 | 217951.6 | 0.93 (0.81, 1.07) | 0.92 (0.80, 1.06) | 0.89 (0.77, 1.03) |
| Quartile 3 (12.59-17.61) | 24594 | 402 | 217486.8 | 0.97 (0.84, 1.11) | 0.94 (0.82, 1.08) | 0.87 (0.75, 1.02) |
| Quartile 4 (≥17.62) | 24602 | 433 | 215901.1 | 1.05 (0.92, 1.20) | 1.01 (0.88, 1.16) | 0.84 (0.69, 1.02) |
| *P* for trend |  |  |  | 0.296 | 0.624 | 0.105 |
| **Non-Small Cell Lung Cancer** |  |  |  |  |  |  |
| Quartile 1 (≤8.90) | 24582 | 371 | 218233.5 | 1.00 (reference) | 1.00 (reference) | 1.00 (reference) |
| Quartile 2 (8.91-12.58) | 24590 | 345 | 217759.5 | 0.93 (0.81, 1.08) | 0.92 (0.80, 1.07) | 0.90 (0.78, 1.05) |
| Quartile 3 (12.59-17.61) | 24523 | 331 | 217126.5 | 0.90 (0.77, 1.04) | 0.87 (0.75, 1.01) | 0.83 (0.70, 0.99) |
| Quartile 4 (≥17.62) | 24530 | 361 | 215542.4 | 0.99 (0.85, 1.14) | 0.95 (0.82, 1.10) | 0.83 (0.67, 1.03) |
| *P* for trend |  |  |  | 0.956 | 0.547 | 0.092 |
| **Small Cell Lung Cancer** |  |  |  |  |  |  |
| Quartile 1 (≤8.91) | 24316 | 48 | 217209.6 | 1.00 (reference) | 1.00 (reference) | 1.00 (reference) |
| Quartile 2 (8.92-12.58) | 24231 | 43 | 215613.9 | 0.90 (0.60, 1.36) | 0.90 (0.60, 1.36) | 0.80 (0.52, 1.22) |
| Quartile 3 (12.59-17.61) | 24263 | 71 | 215858.2 | 1.49 (1.03, 2.15) | 1.47 (1.01, 2.13) | 1.15 (0.76, 1.73) |
| Quartile 4 (≥17.62) | 24241 | 72 | 213984.3 | 1.53 (1.06, 2.20) | 1.51 (1.04, 2.20) | 0.90 (0.54, 1.52) |
| *P* for trend |  |  |  | 0.003 | 0.005 | 0.944 |

a: Adjusted for age (years), sex (male, female) and race (white, non-white).

b: Adjusted for model 1 plus educational level (college below, college graduate, postgraduate), body mass index (kg/m^2^), family history of lung cancer (no, yes, possibly), smoker (never, current or former), drinker (no, yes), aspirin use (no, yes), history of emphysema (no, yes), history of diabetes (no, yes), physical activity (min/week), and energy intake from diet (kcal/day).

**Supplementary Table 4. Association of mono-unsaturated fatty acids with the risk of lung cancer and its subtypes**

|  | **No. of** | **No. of** |  | **Hazard ratio (95% confidence interval)** | | |
| --- | --- | --- | --- | --- | --- | --- |
| **Quartiles of MUFA (g/day)** | **Participants** | **Cases** | **Person-years** | **Unadjusted** | **Model 1^a^** | **Model 2^b^** |
| **Lung Cancer** |  |  |  |  |  |  |
| Quartile 1 (≤14.54) | 24617 | 384 | 219654.0 | 1.00 (reference) | 1.00 (reference) | 1.00 (reference) |
| Quartile 2 (14.55-20.92) | 24647 | 356 | 218530.6 | 0.93 (0.81, 1.08) | 0.92 (0.80, 1.07) | 0.88 (0.76, 1.03) |
| Quartile 3 (20.93-29.64) | 24592 | 439 | 216586.7 | 1.16 (1.01, 1.33) | 1.11 (0.96, 1.28) | 1.02 (0.87, 1.20) |
| Quartile 4 (≥29.65) | 24603 | 463 | 215036.6 | 1.24 (1.08, 1.42) | 1.16 (1.01, 1.34) | 0.99 (0.80, 1.23) |
| *P* for trend |  |  |  | <0.001 | 0.005 | 0.698 |
| **Non-Small Cell Lung Cancer** |  |  |  |  |  |  |
| Quartile 1 (≤14.54) | 24574 | 341 | 219438.6 | 1.00 (reference) | 1.00 (reference) | 1.00 (reference) |
| Quartile 2 (14.55-20.91) | 24560 | 312 | 217946.3 | 0.92 (0.79, 1.08) | 0.91 (0.78, 1.06) | 0.88 (0.75, 1.03) |
| Quartile 3 (20.92-29.63) | 24553 | 376 | 216514.4 | 1.12 (0.97, 1.30) | 1.06 (0.92, 1.24) | 1.00 (0.84, 1.19) |
| Quartile 4 (≥29.64) | 24538 | 379 | 214762.6 | 1.14 (0.98, 1.32) | 1.06 (0.91, 1.24) | 0.95 (0.76, 1.20) |
| *P* for trend |  |  |  | 0.014 | 0.170 | 0.974 |
| **Small Cell Lung Cancer** |  |  |  |  |  |  |
| Quartile 1 (≤14.54) | 24276 | 43 | 218023.1 | 1.00 (reference) | 1.00 (reference) | 1.00 (reference) |
| Quartile 2 (14.55-20.91) | 24292 | 44 | 216522.8 | 1.03 (0.68, 1.57) | 1.03 (0.68, 1.57) | 0.92 (0.60, 1.42) |
| Quartile 3 (20.92-29.63) | 24220 | 63 | 214730.1 | 1.49 (1.01, 2.20) | 1.46 (0.98, 2.17) | 1.18 (0.76, 1.82) |
| Quartile 4 (≥29.64) | 24263 | 84 | 213390.1 | 2.00 (1.39, 2.89) | 1.98 (1.34, 2.91) | 1.29 (0.74, 2.26) |
| *P* for trend |  |  |  | <0.001 | <0.001 | 0.251 |

a: Adjusted for age (years), sex (male, female) and race (white, non-white).

b: Adjusted for model 1 plus educational level (college below, college graduate, postgraduate), body mass index (kg/m^2^), family history of lung cancer (no, yes, possibly), smoker (never, current or former), drinker (no, yes), aspirin use (no, yes), history of emphysema (no, yes), history of diabetes (no, yes), physical activity (min/week), and energy intake from diet (kcal/day).

**Supplementary Table 5. Sensitivity analyses on the association of low-fat diet scores with the risk of lung cancer**

|  | **No. of** | **No. of** | **Hazard Ratio (95% Confidence Interval) by Low Fat Diet Scores ^a^** | | | |  |
| --- | --- | --- | --- | --- | --- | --- | --- |
| **Categories** | **Participants** | **Cases** | **Quartile 1 (≤10)** | **Quartile 2 (11-15)** | **Quartile 3 (16-20)** | **Quartile 4 (≥21)** | ***P* _trend_** |
| Excluded participants with a history of emphysema ^b^ | 96410 | 1476 | 1.00 (reference) | 0.88 (0.77, 1.01) | 0.79 (0.68, 0.91) | 0.78 (0.67, 0.92) | <0.001 |
| Excluded participants with a history of diabetes ^c^ | 91990 | 1526 | 1.00 (reference) | 0.89 (0.78, 1.01) | 0.78 (0.68, 0.90) | 0.75 (0.64, 0.88) | <0.001 |
| Excluded cases observed within the first 2 years of follow-up | 98164 | 1347 | 1.00 (reference) | 0.90 (0.79, 1.03) | 0.76 (0.66, 0.89) | 0.74 (0.62, 0.88) | <0.001 |
| Excluded cases observed within the first 4 years of follow-up | 97836 | 1019 | 1.00 (reference) | 0.92 (0.79, 1.07) | 0.74 (0.62, 0.88) | 0.72 (0.59, 0.88) | <0.001 |
| Further adjusted for pyramid food consumption variables ^d^ | 98459 | 1642 | 1.00 (reference) | 0.91 (0.80, 1.03) | 0.82 (0.70, 0.95) | 0.81 (0.68, 0.98) | 0.010 |
| Further adjusted for dietary constituent intake variables ^e^ | 98459 | 1642 | 1.00 (reference) | 0.92 (0.81, 1.05) | 0.80 (0.70, 0.93) | 0.80 (0.66, 0.96) | 0.004 |
| Repeated analysis in participants with non-missing data | 71492 | 720 | 1.00 (reference) | 0.97 (0.80, 1.17) | 0.84 (0.69, 1.04) | 0.81 (0.64, 1.02) | 0.036 |

a: Hazard ratios were adjusted for age (years), sex (male, female) and race (white, non-white), educational level (college below, college graduate, postgraduate), body mass index (kg/m^2^), family history of lung cancer (no, yes, possibly), smoker (never, current or former), drinker (no, yes), aspirin use (no, yes), history of emphysema (no, yes), history of diabetes (no, yes), physical activity (min/week), and energy intake from diet (kcal/day).

b: Hazard ratio was not adjusted for history of renal comorbidity.

c: Hazard ratio was not adjusted for history of diabetes.

d: Further adjusted for Total fruits (servings/day), Total vegetables (servings/day), Total lean meat (oz./day), Total dairy (servings/day), Added Sugars (tsp/day).

e: Further adjusted for Cholesterol(mg/day), Sodium (mg/day), Potassium (mg/day), Magnesium (mg/day), Calcium (mg/day), Phosphorus (mg/day).
