# Supplementary figures and images for: Adherence to the low-fat diet pattern reduces the risk of lung cancer in American adults aged 55 years and above: a prospective cohort study
Source: J Nutr Health Aging. 2024 Apr 24;28(7):100240. doi: 10.1016/j.jnha.2024.100240 (PMC12433782; doi:10.1016/j.jnha.2024.100240)

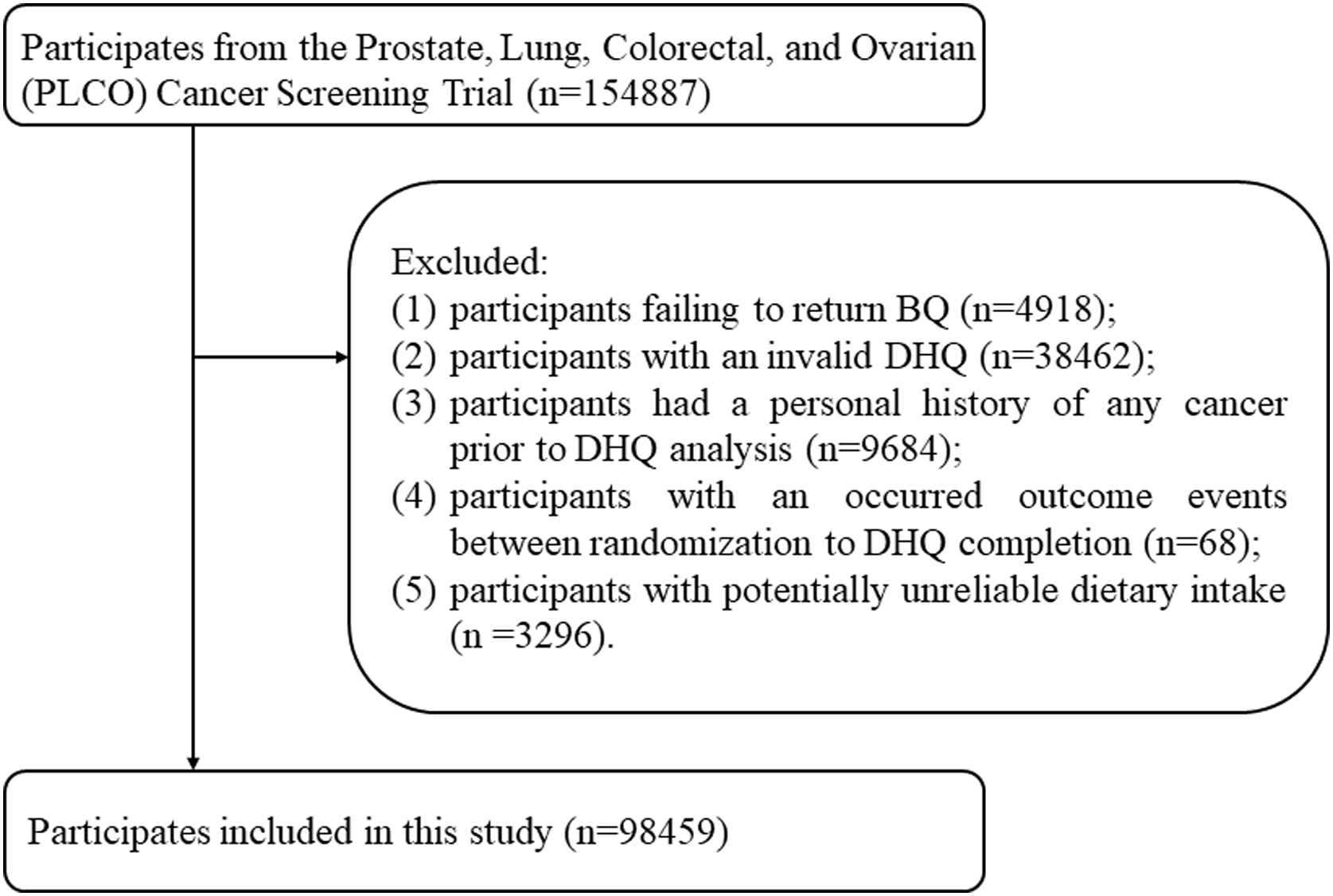

Supplement: Supplementary file 2 — The flow chart of identifying eligible participants. PLCO, Prostate, Lung, Colorectal, and Ovarian; BQ, baseline questionnaire; DHQ, diet history questionnaire. [file mmc2.jpg]

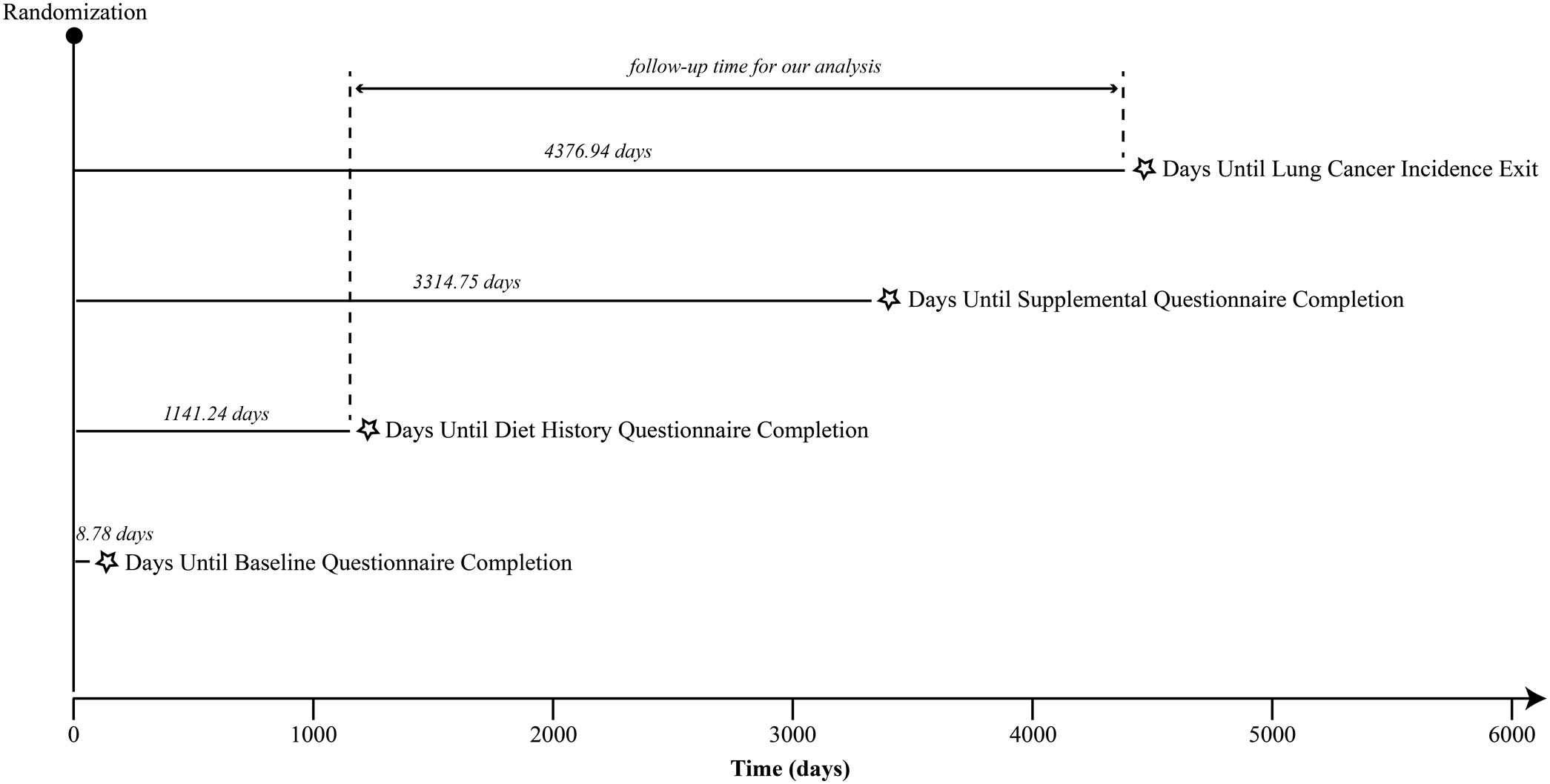

Supplement: Supplementary file 3 — The timeline and follow-up scheme of our study. [file mmc3.jpg]
